# Stunting in Infancy Is Associated with Decreased Risk of High Body Mass Index for Age at 8 and 12 Years of Age^1^,^2^,^3^

**DOI:** 10.3945/jn.116.234633

**Published:** 2016-09-28

**Authors:** Christopher T Andersen, Aryeh D Stein, Sarah A Reynolds, Jere R Behrman, Benjamin T Crookston, Kirk A Dearden, Mary E Penny, Whitney Schott, Lia CH Fernald

**Affiliations:** 4 Division of Epidemiology and; 5 Division of Community Health and Human Development, School of Public Health, University of California, Berkeley, CA;; 6 Department of Epidemiology, Harvard T.H. Chan School of Public Health, Boston, MA;; 7 Hubert Department of Global Health, Rollins School of Public Health, Emory University, Atlanta, GA;; 8 Department of Economics,; 9 Department of Sociology, and; 10 Population Studies Center, University of Pennsylvania, Philadelphia, PA;; 11 Department of Health Science, Brigham Young University, Provo, UT;; 12 IMA World Health, Dar es Salaam, Tanzania; and; 13 Nutrition Research Institute, Lima, Peru

**Keywords:** body mass index, stunting, children, cohort study, Peru

## Abstract

**Background:** Effects of early-life stunting on adiposity development later in childhood are not well understood, specifically with respect to age in the onset of overweight and obesity.

**Objectives:** We analyzed associations of infant stunting with prevalence of, incidence of, and reversion from high body mass index–for-age *z* score (BMIZ) later in life. We then estimated whether associations of infant stunting with BMIZ varied by sex, indigenous status, and rural or urban residence.

**Methods:** Data were collected from 1942 Peruvian children in the Young Lives cohort study at ages 1, 5, 8, and 12 y. Multivariable generalized linear models estimated associations of stunting (height-for-age *z* score <−2) at age 1 y with risk of BMIZ > 1 and BMIZ > 2 prevalence, incidence (moving above a BMIZ threshold between ages), and reversion (moving below a BMIZ threshold between ages) at later ages.

**Results:** After adjustment for covariates, stunting at age 1 y was associated with a lower prevalence of BMIZ > 1 at age 8 y (RR: 0.81; 95% CI: 0.66, 1.00; *P* = 0.049) and 12 y (RR: 0.75; 95% CI: 0.61, 0.91; *P =* 0.004), as well as a lower prevalence of BMIZ > 2 at age 8 y. Stunting was not associated with incident risk of BMIZ > 1 or BMIZ > 2. Stunting was positively associated at age 5 y with risk of reversion from BMIZ > 1 (RR: 1.22; 95% CI: 1.05, 1.42; *P* = 0.008) and BMIZ > 2. We found evidence that the association of stunting with prevalent and incident BMIZ > 1 was stronger for urban children at ages 5 and 8 y, and for nonindigenous children at age 8 y.

**Conclusions:** Stunting predicted a lower risk of prevalent BMIZ > 1 and BMIZ > 2, even after controlling for potential confounders. This finding may be driven in part by a higher risk of reversion from BMIZ > 1 by age 5 y. Our results contribute to an understanding of how nutritional stunting in infancy is associated with BMIZ later in life.

## Introduction

Overweight in children and adolescents is a public health problem in both high-income and low- and middle-income countries (LMICs)^14^. In Latin America, 7.1% of children aged <5 y have a weight-for-height *z* score > 2 (1, 2), and 29–34% of children aged 5–11 y have a body mass index–for-age *z* score (BMIZ) > 1 (3, 4). Overweight or obese children are more likely to be overweight or obese as adults (5), thereby increasing their risk of noncommunicable chronic diseases (6).

Stunting prevalence [height-for-age *z* score (HAZ) <−2] (2) in children <5 y of age in LMICs has declined in recent decades, but 5.9 million children <5 y old (11.6%) were stunted in Latin America and the Caribbean in 2015 (7). Stunting increases mortality risk from infectious diseases during childhood (8), impairs cognitive development (9), and is associated with poorer educational and economic outcomes in adolescence and adulthood (10).

As LMICs undergo demographic, economic, and nutritional transitions, a dual burden of overweight and stunting can occur. A greater prevalence of overweight in stunted children has been reported in cross-sectional studies (11, 12). These findings are not consistent with prospective studies that have found that stunting in early childhood is associated with decreased BMI or body fat in childhood (13, 14), adolescence (15–17), and adulthood (18), and other prospective studies have found null associations (17, 19, 20). To our knowledge, few studies have measured outcomes at >1 follow-up age (13, 14, 17), and none have considered age-specific patterns of overweight incidence and reversion.

In this study we analyzed associations of stunting at age 1 y with the prevalence of, incidence of, and reversion from high BMIZ at ages 5, 8, and 12 y in Peruvian children. Recently, Peru experienced rapid economic growth; it is now in the midst of a nutritional transition. In 2011, the stunting prevalence in children <5 y of age was 19.3%; in 2000, it was 31.4% (21). In 2011, the prevalence of weight-for-height *z* score > 2 (2) in children <5 y of age was 8.8%. In children ages 6–9 y, BMI-for-age ≥85th percentile (4) was 21.5% in 2009–2010 (22). The high prevalence of these 2 conditions makes Peru an opportune setting for analyzing associations between early stunting and later overweight and obesity.

## Methods

### 

#### Data source.

We analyzed data from Peruvian children in the prospective Young Lives cohort study (23). In 2002, 2052 children aged ∼6–18 mo were recruited (round 1). Follow-up data were collected in 2006 when children were ∼5 y old (round 2), in 2009 when children were ∼8 y old (round 3), and in 2013 when children were ∼12 y old (round 4). To simplify reference to each of these rounds of data collection, we will refer to them as ages 1, 5, 8, and 12 y.

Participants were selected through a multistage sampling process. Ten random draws of 20 sentinel sites were conducted from among the 1818 districts in Peru. Consistent with the study’s pro-poor focus, the wealthiest 5% of districts were excluded. From these random draws, one set of 20 sites was selected that best met the study aims of diverse coverage and logistical feasibility. Within selected districts, an initial community was randomly selected as the starting point for recruitment of age-eligible children. Full details of participant recruitment are available elsewhere (24).

#### Anthropometric variables.

Weight and length at age 1 y were measured by 6 supervisors who used calibrated digital balances (Soehnle) with 100-g precision and locally made rigid stadiometers with 1-mm precision. At later ages, measurements were taken by all field staff with the use of similar digital platform balances (with 100-g precision), and standing height was measured with the use of locally made instruments accurate to 1 mm. The staff followed standard WHO procedures for measurement of weight, length, and height. To ensure inter- and intrarater reliability, standard measurement procedures were described in the training manual, and repeat measurements were conducted to ensure accuracy (25). HAZ and BMIZ were calculated according to age-appropriate WHO references (2, 4).

Our predictor of interest was stunting at age 1 y, defined as HAZ <−2. In this sample, HAZ during round 1 was inversely correlated with age in months (26), so children recruited at a younger age were less likely to be classified as stunted. We therefore adjusted all round 1 HAZ measurements to their predicted value at age 12 mo. Applying methodology described elsewhere (26), we calculated the difference between each child’s HAZ and the mean HAZ for children within ±1 mo of the child’s age. This value was then added to the mean HAZ for children aged 11–13 mo.

Outcomes included the prevalence of, incidence of, and reversion from high BMIZ, with the use of thresholds of BMIZ > 1 and BMIZ > 2. The WHO defines overweight and obesity differently for children <5 y of age and those 5–19 y. For children <5 y of age, overweight (including obesity) is defined as BMIZ > 2 and obesity is defined as BMIZ > 3 (2), whereas for children aged 5–19 y, overweight is defined as BMIZ > 1 and obesity is BMIZ > 2 (4). If we adhered to these definitions, children could be considered to develop overweight or obesity without any change in BMIZ. Therefore, for all ages, we consistently defined overweight as BMIZ > 1 and obesity as BMIZ > 2. To maintain clarity, we refer to the exact cutoffs used, rather than the terms overweight and obesity, when referring to the results from this analysis.

**FIGURE 1:**
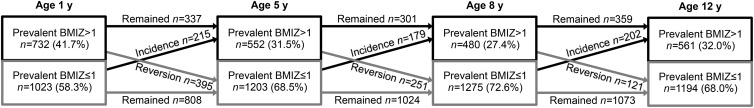
Transitions across BMIZ > 1 threshold in Peruvian children in the Young Lives cohort at ages 1, 5, 8, and 12 y (*n* = 1755). Incidence refers to a transition from BMIZ ≤ 1 at a given age to a BMIZ > 1 at the next age. Reversion refers to a transition from BMIZ > 1 at a given age to a BMIZ ≤ 1 at the next age. BMIZ, body mass index–for-age *z* score.

If a child was above a given BMIZ threshold (i.e., BMIZ > 1 or BMIZ > 2) for the *i*th round, they were defined as a prevalent case for that threshold in the *i*th round. If a child was above the threshold at the *i*th round but below the threshold in the *i*th − 1 round, then we defined that child as an incident case for that threshold at the *i*th round. If a child was below the threshold at the *i*th round but was above the threshold in the *i*th − 1 round, then we defined that child as reverted from that threshold at the *i*th round. These transitions are illustrated graphically for the analyzed sample in **Figure 1**.

#### Covariates.

Covariates were selected for the model on the basis of the causal pathway structure supported by the literature, as well as the data available from the Young Lives study. The statistical significance of a covariate was not a criterion for inclusion in the model, although all covariates were significantly associated with stunting status at age 1 y (**Table 1**). We adjusted for covariates at the child, mother, and household level. At the child level, we adjusted for sex. Child age was not included because it was already adjusted through the BMIZ measure and the adjustment to HAZ in round 1. There was no association between age and BMIZ in any later round. We did not adjust for breastfeeding status because nearly all children (97.7%) had been breastfed for ≥6 mo. We also did not adjust for birth weight because we were interested in stunting at age 1 y as an indicator of chronic malnutrition.

**TABLE 1:** Characteristics of stunted and nonstunted Peruvian children at age 1 y in the Young Lives cohort study^1^

	Not stunted (*n* = 1407)	Stunted (*n* = 535)	*P*^2^
Child characteristics			
HAZ	−0.76 ± 0.84	−2.76 ± 0.63	<0.001
Female	52.4	44.3	0.002
Maternal characteristics			
Height, cm	150.9 ± 5.3	147.7 ± 5	<0.001
BMI, kg/m^2^			<0.001
Normal weight (BMI <25)	56.7	68.8	
Overweight (BMI ≥25 and <30)	33.3	25.0	
Obese (BMI ≥30)	10.0	6.2	
Indigenous	23.5	50.8	<0.001
Completed primary education	77.9	50.3	<0.001
Household characteristics			
≥6 household members	44.6	49.9	0.037
Rural	25.2	53.8	<0.001
Region			<0.001
Coastal	43.1	13.8	
Mountain	42.1	71.4	
Jungle	14.9	14.8	
Wealth index			<0.001
Quintile 1 (lowest)	15.4	30.3	
Quintile 2	16.8	28.0	
Quintile 3	20.0	21.5	
Quintile 4	23.5	10.8	
Quintile 5 (highest)	24.2	9.3	

1 Values are means ± SDs or percentages. HAZ, height-for-age *z* score.

2 Student’s *t* test, Fisher’s exact test, or Pearson’s chi-square test.

Maternal covariates included height and BMI in round 1. Maternal BMI was categorized into 3 mutually exclusive categories: normal [BMI (in kg/m^2^) <25], overweight (BMI ≥25 and <30) and obese (BMI ≥30). There were too few underweight women (BMI <18.5; 1.6%) to include in a separate category, so they were included in the normal BMI category. Mothers whose first language was not Spanish (defined by the language the grandmother spoke to the mother) were classified as indigenous. We also included a binary indicator of whether the mother had completed primary education (≥6 grades of schooling).

Household characteristics included indicators of whether households had ≥6 members or were in rural areas, and geographic regions (coastal, jungle, or mountain). Household wealth was measured with the use of the Young Lives wealth index, which is the mean of 3 composite scores for housing quality, consumer durables, and service access. A detailed description of the wealth index is published elsewhere (24). Wealth was split into nominal quintile indicators for the statistical analysis.

#### Sample size, exclusions, and multiple imputation.

Of the 2052 children initially recruited, 23 were excluded because their ages at recruitment were outside the target range of 6–17 mo. Twenty children were excluded because of documented deaths after baseline, 45 children because of missing HAZ or BMIZ at age 1 y, 11 children because of improbable anthropometric *z* scores (HAZ <−5 or HAZ >3 or BMIZ <−4 or BMIZ >5) (27) during any round, and 11 children because of missing covariate data at age 1 y. This resulted in a sample of 1942 children with complete data at baseline. An additional 187 children were missing BMIZ data at age 5, 8, or 12 y, resulting in 1755 cases with complete follow-up data for analysis.

Details on baseline characteristics of subjects with and without missing follow-up BMIZ data are found in **Supplemental Table 1**. We observed that missingness was associated with some observed covariates, indicating that a complete case analysis might result in biased estimates. To account for potential selection bias (under the assumption of missing at random), we conducted multiple imputation with the use of chained equations to impute missing values of BMIZ (28). Thirty imputations for each missing value were performed (28). Linear regression was used in the multiple imputation procedure to impute predicted values for missing BMIZ at ages 5, 8, and 12 y. All covariates from the main analysis, baseline outcomes, and an indicator variable for the sampling cluster were included in the imputation models.

#### Statistical analysis.

We stratified the data on stunted status at age 1 y and calculated descriptive statistics. We tested differences in covariate values between stunted and nonstunted children at age 1 y, and between those lost to follow-up and those not lost to follow-up, with the use of Fisher’s exact test, Pearson’s chi-square test, and Student’s *t* test. We used generalized linear models with a Poisson distribution, log link, and robust variance (29) to estimate the association between stunting status at age 1 y and the risk of subsequent prevalence of, incidence of, and reversion from BMIZ > 1 or BMIZ > 2. Results from 3 models are reported: *1*) bivariable regressions of outcomes on stunting at age 1 y; *2*) multivariable regressions with controls for potentially confounding covariates with the use of observations with complete outcome data at all ages; and *3*) multivariable regressions adjusted for the same covariates as in the second model, but with imputations for missing outcomes. In regressions of incidence and reversion on stunting status in model 3, the population at risk varied across imputed data sets. To permit analysis, we set the at-risk population across imputed data sets by using mean imputed values for BMIZ at age 5 y and age 8 y to determine whether children were at risk of incidence or reversion at ages 8 and 12 y, respectively. We examined, one interaction at a time, the significance of multiplicative interaction terms between stunting status at age 1 y and sex, indigenous status, and rural or urban status. Statistical significance was considered to be *P* < 0.05. Statistical analyses were conducted with the use of Stata version 13.

#### Ethics.

Ethics committees at the University of Oxford and the Nutrition Research Institute in Lima approved the Peruvian Young Lives study. Parents provided written informed consent in round 1 and verbal reconsent in each subsequent round.

## Results

Over one-quarter of children (27.5%) were stunted at 1 y of age (Table 1). There were large differences between stunted and nonstunted children. The mean HAZ in the stunted group was 2 SDs below that of the nonstunted group. Stunted children were more likely to be male. The mothers of stunted children were shorter, more likely to be indigenous, and less likely to be overweight or to have completed primary education. Stunted children were more likely to live in rural and mountainous regions and were from poorer households. The median age of children in the sample in round 1 was 12 mo (IQR: 8–15 mo).

**FIGURE 2:**
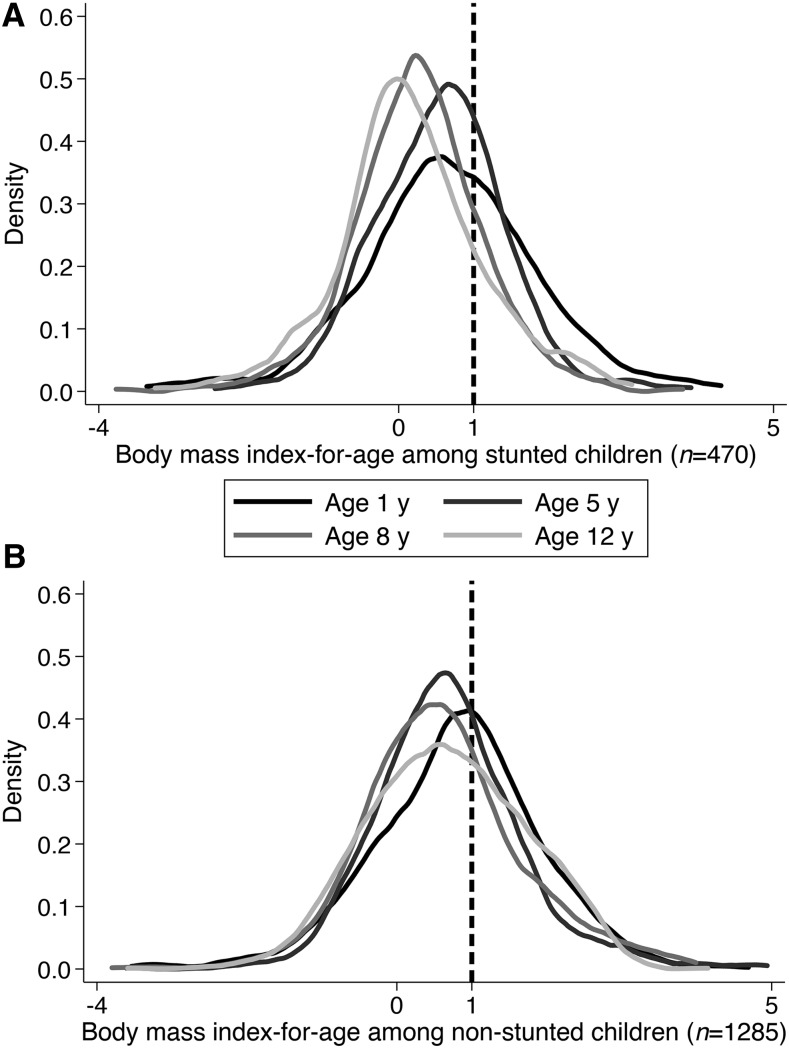
Longitudinal changes in body mass index–for-age distribution in Peruvian children in the Young Lives cohort, stratified by stunted (A) and nonstunted (B) status at age 1 y.

The prevalence of, incidence of, and reversion from BMIZ > 1 in the sample of children with complete follow-up data (*n* = 1755) from 1 to 12 y of age are illustrated in Figure 1. The prevalence of BMI > 1 decreased from 41.7% at age 1 y to 27.4% at age 8 y, but then increased to 32.0% at age 12 y. The incidence of BMIZ > 1 at age 5 y fell from 21.0% of at-risk children (*n* = 1023) to ∼15% at ages 8 and 12 y. Reversion between ages 1 and 5 y was high (54.0%), but was lower in subsequent periods. Kernel densities of the distributions of BMIZ by age, separately for stunted and nonstunted children, are presented in **Figure 2**. The central tendency of the nonstunted distribution did not shift substantially across ages. Distributions for stunted children shifted leftward and became tighter between age 1 y and age 12 y.

The prevalence of BMIZ > 1 was highest in both groups at age 1 y (**Table 2**). By age 5 y, the prevalence of BMIZ > 1 had decreased by >10 percentage points for both groups. At ages 8 and 12 y, the prevalence of BMIZ > 1 of stunted children was approximately one-half that of nonstunted children. Incident BMIZ > 1 was similar for both groups at age 5 y, but stunted children had a lower incidence through ages 8 and 12 y. Reversion was higher for stunted children at ages 5, 8, and 12 y. Similar trends were observed for prevalence of, incidence of, and reversion from BMIZ > 2.

**TABLE 2:** Anthropometric measurement outcomes in Peruvian children from the Young Lives cohort with complete follow-up data^1^

	Not stunted at age 1 y		Stunted at age 1 y		
	At risk, *n*	With outcome, %	At risk, *n*	With outcome, %	*P*^2^
BMIZ > 1					
Prevalence^3^					
Age 1 y	1285	43.1	470	37.9	0.05
Age 5 y	1285	32.9	470	27.4	0.03
Age 8 y	1285	31.0	470	17.4	<0.01
Age 12 y	1285	37.4	470	17.0	<0.01
Incidence^4^					
Age 5 y	731	20.2	292	22.9	0.35
Age 8 y	862	16.8	341	10.0	<0.01
Age 12 y	887	18.6	388	9.5	<0.01
Reversion^5^					
Age 5 y	554	50.4	178	65.2	<0.01
Age 8 y	423	40.2	129	62.8	<0.01
Age 12 y	398	20.6	82	47.6	<0.01
BMIZ > 2					
Prevalence^3^					
Age 1 y	1285	13.1	470	11.7	0.47
Age 5 y	1285	8.2	470	4.0	<0.01
Age 8 y	1285	10.0	470	2.3	<0.01
Age 12 y	1285	11.8	470	4.5	<0.01
Incidence^4^					
Age 5 y	1117	5.8	415	4.1	0.20
Age 8 y	1180	6.2	451	1.8	<0.01
Age 12 y	1156	5.5	459	3.1	0.04
Reversion^5^					
Age 5 y	168	76.2	55	96.4	<0.01
Age 8 y	105	46.7	19	84.2	<0.01
Age 12 y	129	32.6	11	36.4	0.75

1 *n* = 1755. BMIZ, body mass index–for-age *z* score.

2 Student’s *t* test or Fisher’s exact test.

3 For a given age, the child had a BMIZ value at the indicated level.

4 For a given age, the child had a BMIZ value at the indicated level, and at the previous age, the child did not have a BMIZ at this level.

5 For a given age, the child did not have a BMIZ value at the indicated level, and at the previous age, the child did have a BMIZ at this level.

Unadjusted estimates (model 1) show that stunting status at age 1 y was significantly associated with a lower prevalence of BMIZ > 1 at ages 8 and 12 y, and a lower prevalence of BMIZ > 2 at ages 5, 8, and 12 y (**Table 3**). The RR estimates for stunting and prevalent BMIZ > 1 moved further from the null across study rounds, indicating a strengthening association across time. After adjustment for covariates in the sample with complete follow-up data (model 2), magnitudes of associations were reduced. However, there were still statistically significant inverse associations between stunting at age 1 y and prevalent BMIZ > 1 at ages 8 and 12 y. Models that used imputed missing outcome data (model 3) provided similar results.

**TABLE 3:** Stunting at age 1 y as a predictor of BMIZ > 1 and BMIZ > 2 in Peruvian children in the Young Lives cohort^1^

	Age 5 y			Age 8 y			Age 12 y		
	At risk, *n*	RR (95% CI)	*P*	At risk, *n*	RR (95% CI)	*P*	At risk, *n*	RR (95% CI)	*P*
BMIZ > 1									
Prevalence									
Model 1^2^	1755	0.83 (0.71, 0.98)	0.032	1755	0.56 (0.46, 0.70)	<0.001	1755	0.45 (0.37, 0.56)	<0.001
Model 2^3^	1755	0.85 (0.71, 1.01)	0.07	1755	0.79 (0.63, 0.98)	0.029	1755	0.72 (0.59, 0.89)	0.002
Model 3^4^	1942	0.86 (0.73, 1.02)	0.09	1942	0.81 (0.66, 1.00)	0.049	1942	0.75 (0.61, 0.91)	0.004
Incidence									
Model 1^2^	1023	1.13 (0.88, 1.46)	0.34	1203	0.59 (0.42, 0.84)	0.003	1275	0.51 (0.37, 0.72)	<0.001
Model 2^3^	1023	1.06 (0.80, 1.40)	0.68	1203	0.79 (0.55, 1.14)	0.21	1275	0.70 (0.49, 1.00)	0.051
Model 3^4^	1132	1.05 (0.80, 1.37)	0.73	1331	0.84 (0.59, 1.19)	0.33	1412	0.75 (0.53, 1.05)	0.09
Reversion									
Model 1^2^	732	1.29 (1.13, 1.48)	<0.001	552	1.56 (1.31, 1.86)	<0.001	480	2.31 (1.71, 3.11)	<0.001
Model 2^3^	732	1.24 (1.07, 1.45)	0.004	552	1.05 (0.87, 1.25)	0.62	480	0.98 (0.73, 1.33)	0.92
Model 3^4^	810	1.22 (1.05, 1.42)	0.008	611	1.06 (0.88, 1.27)	0.54	530	1.02 (0.75, 1.38)	0.92
BMIZ > 2									
Prevalence									
Model 1^2^	1755	0.49 (0.31, 0.80)	0.004	1755	0.23 (0.13, 0.43)	<0.001	1755	0.38 (0.24, 0.59)	<0.001
Model 2^3^	1755	0.73 (0.45, 1.20)	0.22	1755	0.49 (0.27, 0.88)	0.017	1755	0.74 (0.48, 1.13)	0.16
Model 3^4^	1942	0.84 (0.54, 1.31)	0.45	1942	0.51 (0.29, 0.90)	0.019	1942	0.72 (0.47, 1.10)	0.13
Incidence									
Model 1^2^	1532	0.70 (0.42, 1.19)	0.19	1631	0.29 (0.14, 0.59)	<0.001	1615	0.55 (0.31, 0.97)	0.039
Model 2^3^	1532	0.99 (0.57, 1.72)	0.97	1631	0.54 (0.27, 1.09)	0.09	1615	0.93 (0.52, 1.64)	0.79
Model 3^4^	1695	1.12 (0.69, 1.84)	0.65	1806	0.57 (0.29, 1.13)	0.11	1787	0.89 (0.51, 1.57)	0.70
Reversion									
Model 1^2^	223	1.26 (1.15, 1.40)	<0.001	124	1.80 (1.36, 2.40)	<0.001	140	1.12 (0.49, 2.54)	0.79
Model 2^3^	223	1.16 (1.04, 1.29)	0.007	124	1.33 (0.97, 1.81)	0.07	140	0.94 (0.41, 2.16)	0.88
Model 3^4^	247	1.16 (1.04, 1.29)	0.010	136	1.40 (0.99, 1.96)	0.06	155	0.96 (0.42, 2.18)	0.92

1 Results for generalized linear models with a Poisson distribution, log link, and robust variance. BMIZ, body mass index–for-age *z* score.

2 Bivariate model predicting overweight or obesity on the basis of stunting.

3 Adjusted for child sex; maternal height, BMI, indigenous status, and primary education; and number of household members, rural location, geographic region, and wealth quintile.

4 Adjusted for the same covariates in model 2, but uses multiply imputed data in addition for missing outcomes.

Stunting at 1 y of age was inversely associated with incident BMIZ > 1 and incident BMIZ > 2 at ages 8 and 12 y, but not at age 5 y (model 1). However, models 2 and 3 found null associations.

Stunting at age 1 y was positively associated with reversion from BMIZ > 1 at ages 5, 8, and 12 y, and was positively associated with reversion from BMIZ > 2 at ages 5 and 8 y (model 1). In models 2 and 3, we found that stunting at age 1 y was positively associated with reversion from BMIZ > 1 and BMIZ > 2 at age 5 y, but was not significantly associated with reversion for either threshold at ages 8 and 12 y.

Significant interactions by rural compared with urban status existed for prevalence and incidence outcomes at ages 5 and 8 y, and for indigenous status at age 8 y (**Table 4**). There were no other statistically significant interaction terms. For the significant interactions, point estimates for the RR were <1 for urban and nonindigenous children, and >1 for rural and indigenous children. However, although there were significant interactions, several of the stratum-specific estimates were not significantly different from 1.

**TABLE 4:** Modification of association of stunting at age 1 y with the prevalence of, incidence of, and reversion from BMIZ > 1 by sex, indigenous status, and rural region in Peruvian children in the Young Lives cohort^1^

	Age 5 y		Age 8 y		Age 12 y	
	Association of stunting^2^	*P*^3^	Association of stunting^2^	*P*^3^	Association of stunting^2^	*P*^3^
Prevalent BMIZ > 1						
Sex		0.43		0.39		0.97
M	0.82 (0.67, 1.01)		0.76 (0.58, 0.99)		0.75 (0.58, 0.96)	
F	0.94 (0.72, 1.22)		0.90 (0.66, 1.24)		0.74 (0.55, 1.01)	
Indigenous status		0.61		0.022		0.71
Nonindigenous mother	0.83 (0.67, 1.04)		0.67 (0.50, 0.88)		0.76 (0.61, 0.95)	
Indigenous mother	0.91 (0.70, 1.18)		1.10 (0.79, 1.55)		0.70 (0.46, 1.06)	
Residence area		0.047		0.003		0.20
Urban	0.74 (0.58, 0.94)		0.64 (0.48, 0.84)		0.81 (0.66, 1.00)	
Rural	1.04 (0.81, 1.34)		1.29 (0.88, 1.89)		0.58 (0.37, 0.93)	
Incident BMIZ > 1						
Sex		0.46		0.18		0.92
M	0.98 (0.71, 1.35)		0.68 (0.42, 1.11)		0.76 (0.50, 1.16)	
F	1.19 (0.78, 1.83)		1.08 (0.67, 1.71)		0.73 (0.44, 1.23)	
Indigenous status		0.57		0.014		0.96
Nonindigenous mother	0.98 (0.69, 1.39)		0.55 (0.32, 0.95)		0.74 (0.50, 1.10)	
Indigenous mother	1.14 (0.76, 1.71)		1.41 (0.83, 2.39)		0.76 (0.40, 1.43)	
Residence area		0.040		0.011		0.13
Urban	0.79 (0.53, 1.17)		0.59 (0.36, 0.96)		0.89 (0.62, 1.28)	
Rural	1.36 (0.94, 1.97)		1.60 (0.88, 2.94)		0.50 (0.25, 0.98)	
Reversion from BMIZ > 1						
Sex		0.20		0.74		0.34
M	1.35 (1.08, 1.68)		1.03 (0.81, 1.31)		0.93 (0.63, 1.36)	
F	1.13 (0.94, 1.35)		1.09 (0.86, 1.39)		1.19 (0.78, 1.81)	
Indigenous status		0.86		0.49		0.37
Nonindigenous mother	1.24 (1.02, 1.50)		1.12 (0.88, 1.43)		0.82 (0.43, 1.57)	
Indigenous mother	1.20 (0.97, 1.50)		0.99 (0.78, 1.27)		1.14 (0.81, 1.60)	
Residence area		0.85		0.21		0.51
Urban	1.24 (1.02, 1.50)		1.21 (0.90, 1.63)		0.89 (0.49, 1.60)	
Rural	1.20 (0.96, 1.50)		0.96 (0.79, 1.18)		1.11 (0.78, 1.59)	

1 Values are RRs (95% CIs), *n* = 1942. One multiplicative interaction term was included separately in the model for each covariate presented. Models include covariates and imputed outcomes. BMIZ, body mass index–for-age *z* score.

2 Results for generalized linear models with a Poisson distribution, log link, and robust variance.

3 *P* value on multiplicative interaction term.

## Discussion

This study found that 4 of 10 Peruvian children had a BMIZ > 1 at age 1 y. As children aged, the prevalence of BMIZ > 1 and BMIZ > 2 decreased in both stunted and nonstunted (at age 1 y) children, with greater reductions in stunted children. This difference cannot be fully explained by controlling for potential confounders at ages 8 and 12 y. On the other hand, in bivariate analyses, early stunting was associated with a reduced incidence of BMIZ > 1 and BMIZ > 2 at ages 8 and 12 y, but these differences disappeared with adjustment. Decreases in prevalent high weight partially appeared to be due to significantly greater reversion associated with stunting at age 5 y. There is evidence that associations of stunting with prevalent and incident BMIZ > 1 were stronger for urban children at ages 5 and 8 y, and for nonindigenous children at age 8 y. Prior longitudinal studies indicated that stunting in early childhood was associated with decreased BMI or body fat throughout later life (13–18). Our study contributes to existing longitudinal data because, to our knowledge, it is the first to investigate associations of stunting in infancy with the incidence of or reversion from overweight and obesity in childhood. This informs an understanding of when high BMIZ develops or subsides during the course of childhood.

Although the direction of the association between stunting at age 1 y and BMIZ > 1 and BMIZ > 2 outcomes was consistent, the magnitude varied. Stunting at age 1 y was associated with a 19% reduction in the risk of prevalent BMIZ > 1 at age 8 y and a 25% reduction in the risk of prevalent BMIZ > 1 at age 12 y. The association was stronger for the risk of prevalent BMIZ > 2 at age 8 y (49% reduction), but similar for the risk of prevalent BMIZ > 2 at age 12 y (28% reduction). The probability of reversion from BMIZ > 1 at age 5 y was 22% higher in stunted children, whereas the probability of reversion from BMIZ > 2 at age 5 y was ∼16% higher.

Rising obesity rates in stunted children in developing countries are a concern (30). Our results indicate that although some stunted Peruvian children have a high BMIZ, there is no evidence of increased high BMIZ status in stunted children compared with nonstunted children. A potential pathway through which stunting in infancy may influence subsequent high BMIZ is delay of the onset of puberty (31). Puberty is associated with an increase in BMI, so a relatively late onset of puberty may result in a decrease in the prevalence of overweight at age 12 y. Indeed, in our sample, 38% of children stunted at 1 y of age and 45% of children not stunted at 1 y of age (Pearson’s chi-square *P* = 0.02) had demonstrated signs of puberty at age 12 y (i.e., voice change and facial hair for boys, and onset of menses for girls), suggesting that puberty is a pathway that merits exploration in future research. However, the differing prevalence of signs of puberty between stunted and nonstunted children would not explain the decrease in prevalent overweight at age 8 y that was associated with infant stunting, which suggests that there are potentially other pathways operating here.

Some studies conclude that stunting may contribute to greater adiposity from impaired fat metabolism and higher fasting respiratory quotients (32–36). Although our study does not indicate an increased overall risk of higher BMIZ, we did find differences between urban and rural children at ages 5 and 8 y, and between indigenous and nonindigenous children at age 8 y. RRs of prevalent and incident BMIZ > 1 for rural and indigenous children were >1, but not significantly. Another study in Peru found that in urban lowland children, length was positively associated with BMI (37), which is consistent with our finding that infant stunting is associated with reduced prevalence and incidence of BMIZ > 1 at age 8 y in urban children. The authors of that study suggested that differences between rural and urban populations may arise from environments that present different opportunities for catch-up growth and the accrual of adipose tissue. Understanding potential effect modification may therefore be important for determining the generalizability of findings on the effects of stunting on overweight.

The multiple tests of interaction we conducted increased the probability of erroneously rejecting the null hypothesis. If we applied a Bonferroni correction to adjust for the 27 tests conducted in Table 3, we would achieve a conservative *P* value cutoff for significance at *P* = 0.0019. Using this adjusted cutoff, we observe that none of the interactions met this threshold of statistical significance. However, the consistent trend of significant interactions (*P* < 0.05) for urban compared with rural status at ages 5 and 8 y for high BMIZ prevalence and incidence outcomes suggests to us that this result may not be due to chance alone. Likewise, we observed significant interactions for α = 0.05 by indigenous status for both prevalence and incidence of BMIZ > 1 at age 8 y. We view these results as suggestive of potential interactions that merit further inquiry in future research.

Given the longitudinal nature of this study, adhering to the WHO cutoff of BMIZ > 2 for overweight in children <5 y of age and the cutoff of BMIZ > 1 for children ≥5 y of age would result in an artificial increase in the incidence and prevalence of overweight between ages 1 and 5 y. This could erroneously give the impression that the distribution of child BMIZ was shifting to the right as children aged. However, the opposite is the case, as demonstrated in Figure 2. All analyses therefore should be interpreted with respect to the indicated BMIZ cutoff, and not as “overweight” or “obesity” according to WHO definitions.

Child age was inversely associated with HAZ in round 1. To address potential bias introduced by the fact that younger children were underrepresented in the stunted population, we calculated the predicted HAZ for children at 12 mo of age. This method assumed that children of a given age maintained their relative position in the HAZ distribution at age 12 mo. We cannot assess the plausibility of this assumption with the Young Lives data. However, the Guatemalan Institute for Nutrition in Central America and Panama longitudinal study, which has been used in influential studies of the longer-run effects of early-life undernutrition (38), does have data on HAZ for the same children at ages 6, 12 and 18 mo. In that study, the correlation between HAZ at ages 6 mo and 12 mo was 0.84, and at ages 12 and 18 mo was 0.89 (JR Behrman, unpublished results, 2016). If similar correlations hold for the Young Lives data, our procedure yields good but not perfect estimates of HAZ at age 12 mo even for the youngest and oldest children in the sample in round 1. To further explore the plausibility of our estimates, we conducted sensitivity analyses (results not shown) by conducting our analyses on the subset of children who were aged 11 and 13 mo in round 1. We found that estimates of the association between stunting and subsequent BMIZ > 1 and BMIZ > 2 outcomes tended to be somewhat further from the null, indicating that any measurement error in our round 1 assessments of stunting made our estimates conservative.

Procedures for the measurement of anthropometric status were standardized and performed 2 times by the same data collector to ensure data validity. However, a slight degree of random measurement error is inevitable. Such random variation is not likely to differ systematically within the cohort, so any bias introduced from mismeasurement would be toward the null. As a result, our findings again would be a conservative estimate of the true association between infant stunting and subsequent high BMIZ.

Approximately 10% of the eligible observations in our data set were missing BMIZ data at age 5, 8, or 12 y. Children missing outcome data were significantly different from those with full covariate data across several characteristics. Therefore, complete case analyses may be biased. To account for this potential bias, we conducted multiple imputation of missing outcomes and report estimates for this analysis. Analyses that used complete cases and those that used multiple imputation returned nearly identical results.

This study contributes to the body of evidence related to whether early-life stunting promotes physiologic changes that influence adiposity later in life. Our evidence suggests that this is not the case for our population, even after controlling for potential confounders, and that stunting is instead associated with reduced prevalence of overweight later in childhood. Although the distribution of BMIZ at age 1 y is similar for stunted and nonstunted children, the distributions diverge throughout childhood, with a greater decline in BMIZ observed for children stunted at 1 y of age. More research is needed to understand the physiologic mechanisms that underlie the relation between early-life stunting and later weight gain, as well as what might explain the observed effect modification by rural and indigenous status.

## Footnotes

14 Abbreviations used: BMIZ, body mass index-for-age *z* score; HAZ, height-for-age z score; LMIC, low- and middle-income country.
